# A Telehealth-Based Cognitive-Adaptive Training (e-OTCAT) to Prevent Cancer and Chemotherapy-Related Cognitive Impairment in Women with Breast Cancer: Protocol for a Randomized Controlled Trial

**DOI:** 10.3390/ijerph19127147

**Published:** 2022-06-10

**Authors:** Ángela González-Santos, Maria Lopez-Garzon, Carmen Sánchez-Salado, Paula Postigo-Martin, Mario Lozano-Lozano, Noelia Galiano-Castillo, Carolina Fernández-Lao, Eduardo Castro-Martín, Tania Gallart-Aragón, Marta Legerén-Álvarez, Rocío Gil-Gutiérrez, Lydia Martín-Martín

**Affiliations:** 1 Department of Physiotherapy, Faculty of Health Sciences, University of Granada, 18011 Granada, Spain; angelagonzalez@ugr.es (Á.G.-S.); paulapostigo@ugr.es (P.P.-M.); mlozano@ugr.es (M.L.-L.); noeliagaliano@ugr.es (N.G.-C.); carolinafl@ugr.es (C.F.-L.); eduardoc@ugr.es (E.C.-M.); lydia@ugr.es (L.M.-M.); 2 “CUIDATE” Research Group (BIO-277), University of Granada, 18011 Granada, Spain; rogilgu@ugr.es; 3 Instituto de Investigación Biosanitaria ibs.GRANADA, 18012 Granada, Spain; 4 Hospital Universitario Virgen de las Nieves, 18014 Granada, Spain; carmen.sanchezsalado@gmail.com; 5 Sport and Health University Research Institute (iMUDS), University of Granada, 18011 Granada, Spain; 6 Hospital Universitario San Cecilio, 18016 Granada, Spain; tania_ga84@hotmail.com (T.G.-A.); marta.legeren@gmail.com (M.L.-Á.); 7 Department of Nursing, Faculty of Health Sciences, University of Granada, 18011 Granada, Spain

**Keywords:** breast neoplasms, cognitive impairment, occupational therapy, telerehabilitation

## Abstract

Background: Many women with breast cancer experience a great number of side effects, such as cognitive impairment, during and after chemotherapy that reduces their quality of life. Currently, research focusing on the use of non-pharmacological, and specifically telehealth interventions to prevent or mitigate them has been insufficient. Methods: This protocol describes a randomized controlled trial aimed at studying the preventive effects of a videoconferenced cognitive-adaptive training (e-OTCAT) program (ClinicalTrials.gov NCT04783402). A number of 98 eligible participants will be randomized to one of the following groups: (a) the experimental group receiving the e-OTCAT program during 12 consecutive weeks since the beginning of chemotherapy; and (b) the control group receiving and educational handbook and usual care. The primary outcome will be the cognitive function. Secondary measures will be psychological distress, fatigue, sleep disturbance, quality of life and occupational performance. The time-points for these measures will be placed at baseline, after 12 weeks and six months of post-randomization. Conclusion: This trial may support the inclusion of multidimensional interventions through a telehealth approach in a worldwide growing population suffering from breast cancer, emphasizing the prevention of cognitive impairment as one of the side effects of cancer and its treatments.

## 1. Introduction

Breast cancer (BC) and its treatments are accompanied by multiple consequences that need to be addressed [1], one of them being cognitive impairment [2].

The terminology to refer to this problem has evolved from *chemobrain* [3] to *cancer and/or chemotherapy related cognitive impairment* (CRCI) [4], after discovering that there are other factors related to cancer and its treatment that influence cognitive decline besides chemotherapy [5]. Nevertheless, patients undergoing chemotherapy have a higher chance of experiencing cognitive impairment [5]. The CRCI consists of an affection of the cognitive domains of attention, working memory and executive functions [6] that have an impact on patients’ daily lives and quality of life [7]. The incidence of CRCI among BC patients is approximately 33% in those treated with chemotherapy [8]. Relating to its prevalence, 60–70% of women who have received this treatment report cognitive complaints during and immediately after chemotherapy [9,10].

Despite its frequency, the responsible mechanisms of CRCI are not yet entirely clear, but they appear to be multifactorial. Aging is a major risk factor for cognitive decline and may enhance the neurotoxic effects of chemotherapy [11]. Other demographical and lifestyle factors are potential contributors of CRIC such as education, socioeconomic status, menopausal status, physical activity level, smoking or alcohol consumption and general health condition [11]. Psychological distress [12], fatigue [13], or sleep disorders [14], as a result of the treatment and the cancer itself, might also play a role in the onset and the course of cognitive decline. Meanwhile, some chemotherapeutic agents such as anthracyclines may induce oxidative stress, pro-inflammatory cytokine levels, and consequently brain injury and CRCI [15]. Taxane drugs used in other chemotherapy regimens have the ability to cross the blood brain barrier and directly induce central neurotoxicity, and therefore CRCI, through neuroinflammation, abnormal neurogenesis and neural apoptosis [16]. In this sense, we are facing a growing population with a high risk to suffer from a lengthy alteration in these areas.

On the other hand, telehealth approaches have become a promising and practiced more frequently among healthcare disciplines [17]. The delivery of remote interventions through digital technology has the potential to reduce economic, time-related and geographical constraints in healthcare systems [18]. Furthermore, telehealth is not a new perspective in cancer patients. Technological support such as web- or telephone-based interventions has been used in cancer rehabilitation, obtaining significant positive outcomes to ameliorate CRCI [19,20,21] and achieve an occupational performance as close as possible to that prior to cancer treatment [22]. This approach includes asynchronous or real-time interventions that might be combined to improve the quality of care. In addition, the current COVID-19 pandemic highlights the need for remote interventions to ensure supportive care for at-risk populations such as cancer patients [23].

We propose the e-OTCAT program, taking up the recommendation of a recent meta-analysis suggesting the testing of the effectiveness of interventions targeted to cognitive impairment in a preventive setting for BC patients scheduled for neurotoxic chemotherapy [24], as well as the potential of occupational therapy and the advantages of a telehealth approach. It will involve a telehealth cognitive-adaptive training to prevent CRCI in women with BC undergoing neurotoxic chemotherapy. The e-OTCAT program has been created to work in combination with the effects of cognitive training to enhance brain plasticity and to address other co-existing factors during this period such as psychological distress and fatigue, as well as occupational performance and quality of life.

We hypothesize that women with BC performing the e-OTCAT program simultaneously to the first course of chemotherapy will report less CRCI, better quality of life with other clinical variables, and better occupational performance than those receiving the usual care.

The main objective will be to study the preventive effects of the e-OTCAT program on CRCI, comparing the differences on the cognitive function between two groups of women with BC undergoing chemotherapy: the experimental group (EG) performing the e-OTCAT program for 12 weeks, starting at the same time as they begin the first course of chemotherapy, and the control group (CG) receiving the usual care.

Secondary objectives will be to investigate the immediate and medium-term effects of the e-OTCAT program on cognitive function, psychological distress, fatigue, sleep quality, quality of life and occupational performance in women with BC undergoing chemotherapy.

## 2. Materials and Methods

### 2.1. Study Design

**Figure 1:**
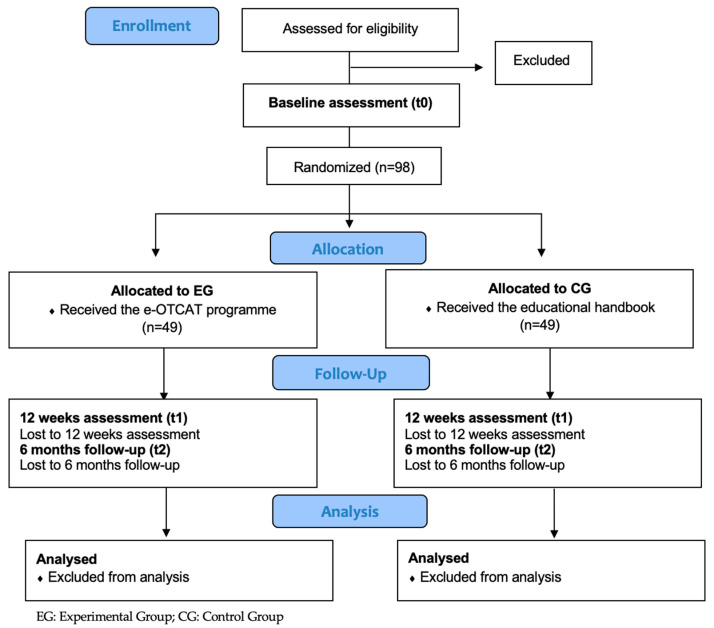
Flow diagram of the randomized controlled trial showing the recruitment of patients.

We propose to carry out a single blinded randomized controlled trial. This study will be conducted by the CUIDATE research group (BIO-277 group) from the University of Granada. The trial is based in accordance with the Template for Intervention Description and Replication (TIDieR) checklist [25] and registered in ClinicalTrials.gov NCT04783402. Figure 1 shows the flow diagram of the study participants.

### 2.2. Participants

Participants will be recruited from the University Hospital of San Cecilio and the University Hospital of Virgen de las Nieves (Granada, Spain), referred by the oncologists and nurses in charge. Eligible women with BC scheduled to receive chemotherapy will be recruited if they meet the inclusion criteria as follows: (a) female BC who are going to start adjuvant or neoadjuvant chemotherapy, (b) stage I-III of BC, (c) age range 18–70 years old, (e) must be able to speak, read and write Spanish well to complete written and verbal assessment, neurocognitive tests and study sessions, and (f) must have basic phone skills. Women will be excluded if they have: (a) a history of cognitive, mental and/or physical disorder that may make participation in the study difficult, the completion of assessments and the performance of intervention i.e., Alzheimer disease, Parkinson’s disease, multiple sclerosis, schizophrenia, bipolar disorder, etc.); (b) a prior history of cancer or secondary diagnosis of cancer, (c) a prior history of chemotherapy, or (d) hearing or visual deficit.

### 2.3. Procedure

**Figure 2:**
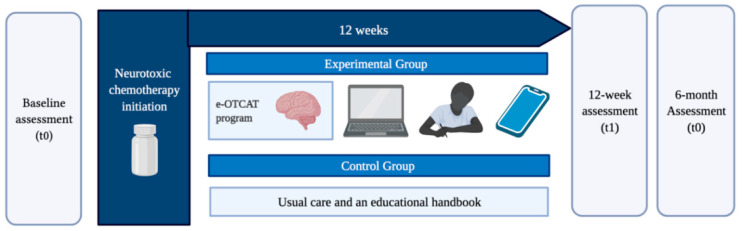
Time-points of the study. Created by Biorender.com.

After checking the eligibility of participants and receiving detailed information from the study, women will be scheduled by a researcher for baseline assessment in the days prior to the start of chemotherapy treatment. Assessments will be carried out in the CUIDATE group facility by a researcher not involved in the implementation of the study intervention. Assessments will be developed at three time-points: at baseline just before chemotherapy (t0), 12-weeks (t1) and six-months (t2) post-randomization. The e-OTCAT program will be conducted during 12 consecutive weeks starting at the same time that women begin chemotherapy treatment. Figure 2 shows the time-points of the study.

Ethical approval has been obtained from the Committee of Biomedical Research Ethics of Andalucía (Granada, Spain) and it adheres to the principles of the Helsinki Declaration (2013 version) [26]. All eligible participants will be informed about the study orally and in writing and they will sign the informed consent if they agree to participate.

### 2.4. Outcome Measures

#### 2.4.1. Primary Outcome

##### Cognitive Function

The Functional Assessment of Cancer Therapy-Cognitive Function (FACT-Cog) version 3 [27] will be used to assess subjective cognitive function. The FACT-Cog comprises 37 questions to be scored from 0 (“never” or “nothing” to 4 “several times a day” or “very much”), and grouped into four subscales: perceived cognitive impairment, perceived cognitive abilities, impact of perceived cognitive impairment on QoL and comments from others about cognitive performance. Higher scores indicate better cognitive function. Cronbach’s α values for BC patients range from 0.71 to 0.93 [28]. The Spanish version of the scale will be used.

#### 2.4.2. Secondary Outcomes

##### Other Cognitive Function Measures

The Trail Making Test (TMT) will be used to assess psychomotor speed, attention skills (TMT-A), flexibility, attentional set-shifting and mental tracking (TMT-B) [29]. Smaller scores mean better results. The B/A ratio will be calculated as a measure of executive control. The TMT is recommended by the International Cancer and Cognition Task Force (ICCTF) to measure cognitive function in cancer patients for its adequate psychometric properties [30].

Two indexes of the Wechsler Adult Intelligence Scale (WAIS-IV) will be used: the Working Memory Index (WMI), comprising digit span and arithmetic subtests, and the Processing Speed Index (PSI), comprising symbol search and coding subtests [31]. WMI and PSI have high reliability, with a Cronbach’s α of 0.94 and 0.90, respectively [32]. The Spanish version of the subtests comprising the scale will be used.

##### Psychological Distress

The Hospital Anxiety and Depression Scale (HADS) will be used to assess anxiety and depression [33]. A total score of 8 or more in both subscales indicates a possible depression/anxiety condition. The HADS has been validated in BC patients. Reliability values for the full scale and the anxiety and depression subscales are 0.90, 0.85 and 0.84, respectively [34]. We will use its Spanish validation for this study.

##### Fatigue

The Piper Fatigue Scale-Revised (PFS-R) will be used to assess cancer-related fatigue grouped into four areas: behavioural, emotional, sensorial and cognitive [35]. Higher scores indicate the greater impact of fatigue. The scale has a Cronbach’s α from 0.87 to 0.89 [36]. The Spanish version of the PFS-R will be applied.

##### Sleep Quality

The Pittsburgh Sleep Quality Index (PSQI) will used to measure seven subjective domains of sleep: quality, latency duration, efficiency, disturbance, medication use and daytime dysfunction [37]. A total score of ≤5 indicates a “good sleeper”, while total scores of >5 indicate poor sleep quality. The PSQI has demonstrated a good reliability in the BC population [38]. The PSQI is validated in Spanish, so this version will be used.

##### Quality of Life

The European Organization for Research and Treatment of Cancer Quality of Life Questionnaire Core 30 (EORTC QLQ-C30) version 3.0 was used. The EORTC QLQ-C30 is grouped into functional scales (physical, role, cognitive, emotional and social), symptoms scales (fatigue, nausea and vomiting, pain, dyspnoea, sleep disturbance, appetite loss, constipation, diarrhoea and financial impact), and a global health status/quality of life scale [39]. Higher scores indicate a greater level of functioning, more symptoms and greater quality of life. The overall quality of life subscale has a Cronbach’s α of 0.89 [40]. The Spanish version of the EORTC QLQ-C30 scale will be applied.

##### Occupational Performance

The Canadian Occupational Performance Measure (COPM) will be used to assess daily activity performance and satisfaction with daily activities [41]. A mean total score for performance and satisfaction is obtained, and higher scores indicate greater performance and satisfaction. The COPM has great test-retest, interrater reliability, and convergent and divergent validity values [42]. This tool has been translated into the Spanish language, so this version will be used.

### 2.5. Intervention

Eligible women will be randomized to one of the following groups: (a) EG: receiving the e-OTCAT program; or (b) CG: receiving the usual care. Procedures for each group are described as follows:

**For EG**: Participants will receive the e-OTCAT program during 12 consecutive weeks, starting at the same time that they begin the first course of adjuvant or neoadjuvant chemotherapy. Each week will include two individualized sessions via videoconference using web conferencing software, conducted by a trained occupational therapist. Sessions will be organized as follows (see Table 1 for more detailed information):

- ***Cognitive training*** sessions will take place once a week (12 sessions) by videoconference (30′), where the participant will perform exercises to strengthen cognitive function in terms of attention, memory and processing speed. Besides this weekly session, each participant must complete a weekly series of cognitive training exercises at home. These will consist of an exercise using a handbook and paper and pencil and the mobile application of NeuroNation©, a German brain-training software company. We will encourage participants to complete a minimum of 20′ minutes per week of each homework modality.
- ***Adaptive training*** sessions will take place also once a week since the second week to the second to last (10 sessions) through the videoconference, and it will be based on cognitive-behavioural techniques. Participants will train in the following areas: psychoeducation concerning stress management, relaxation techniques (diaphragmatic breathing, progressive muscle relaxation and differential relaxation), problem-solving training, and energy conservation techniques. After each session of adaptive training, participants will be motivated to practice the strategy seen in the session during the following days of the week until the next adaptive training session.

The therapist will monitor the adherence to homework cognitive training activities (mobile app and handbook) and adaptive strategies training through daily reports from the mobile app and self-registration for each activity.

**For CG**: Participants who are part of CG will receive standard care and an educational handbook containing general information about the most frequent side-effects of cancer and its treatment, most specifically about CRCI, psychological distress, cancer-related fatigue, sleep problems and lymphedema of the upper limbs. For ethical reasons, participants pertaining at CG could receive the e-OTCAT program when their participation in the study is completed.

### 2.6. Sample Size

The sample size for cognitive function was calculated based on a similar previous study using FACT-Cog version 3.0 [43]. Assuming an alpha error of 0.05, a power of 85% and a medium effect size (d = 0.58) based on the results of the reference study, we need a sample of 44 participants for each group (G*Power v.3.1). In order to compensate for a possible 10% dropout rate [44], we will recruit a total of 98 participants (49 per group).

### 2.7. Randomization and Blinding

We will use a computer-generated number (Epidat 4.2 software, Xunta de Galicia, A Coruña, Spain) to assign each participant to the EG or CG. After completion of baseline assessment (t0), randomization will be applied by an independent researcher not involved in the study. The researcher will communicate in which group the participant has been allocated to the researcher responsible for the program (EG or CG). The researcher in charge of assessments at all time points will be blind to the intervention group. To avoid contamination of the CG and EP, the CG will be unaware of the actions carried out in detail in the EG. In addition, for both groups it will be recorded whether during the course of the study they have received any additional interventions that may influence the outcome variables.

### 2.8. Statistical Analysis

The SPSS 25 software (IBM Statistic Program for the Social Sciences SPSS Statistic, Corp., Armonk, NY, USA) will be used for all data analyses. In all analyses, *p* < 0.05 values will be considered statistically significant. A blind researcher will perform the statistical analysis.

Descriptive statistics will be used to examine the sociodemographic and clinical characteristics of the sample. Data will be expressed as mean ± standard deviation (SD) or means (95% CI) for continuous, and as number or percentages for categorical variables. We will check the normal distribution of variables with the Kolmogorov-Smirnov test and visual inspection. Comparison between the two groups of the study (EG and CG) for baseline demographic data will be done with Student’s *t*-test for continuous, or a Chi-square test for categorical variables. Their non-parametric homologues, i.e., the Mann-Whitney U and Fisher’s exact test, will be used as appropriate.

Bivariate correlations will be used to explore the role of different variables that have been shown to determine cognitive performance in BC patients. Age and educational level will be considered as variables to be controlled for data analysis. Differences in cognitive function of participants receiving vs. not receiving the e-OTCAT program, as well as differences within-groups, will be calculated with a generalized linear mixed model. Substitution by means will be undertaken to treat the possible missing data. All analysis will be “intention-to-treat”. Calculation of the intergroup effect size (using Cohen’s d) will be performed to provide magnitude changes.

## 3. Discussion

Knowledge about effective interventions targeted to CRCI in BC patients is still inconsistent [45]. Along these lines, the e-OTCAT program has been developed to study the preventive effects of a 12-week telehealth cognitive-adaptive training based on occupational therapy on CRCI in women with BC undergoing chemotherapy.

This trial is a pioneer in addressing CRCI at the beginning of chemotherapy, when the negative impact on cognitive function is not yet fully developed. A similar pilot study carried out by Park et al. [44], implemented a 12-week phone-based compensatory cognitive training to help BC patients undergoing chemotherapy learned skills to self-manage and cope with CRCI. Their program showed positive preliminary results in improving objective and subjective cognitive functions. However, the lack of randomization and other methodological issues limits their conclusions. To our knowledge, there are no other studies aimed to achieve the same preventive goal with a similar intervention prior to the beginning of chemotherapy. Nevertheless, there is an emergent interest to develop non-pharmacological programs targeted to prevent neurocognitive impairments in at-risk populations from cognitive impairment, such as Alzheimer disease. Multidomain programs containing cognitive training and other lifestyle interventions (psychoeducation, physical exercise, diet, etc.) are highlights to successfully address the multifactorial origin of cognitive impairment across different life stages.

In addition, the telematic modality of these programs is considered a good option to enhance cognitive function an in at-risk population [46]. Considering the recent boom in the use of telehealth [23], the proposed telematic and individual approach may be a way to provide easy access to care for this population, reducing several barriers and adapting to the possibilities and circumstances of each patient.

There are some limitations in our study. One of them is the risk of adherence bias, although we intended to take advantage of the telematic and playful nature of the proposed activities to motivate the participants. In addition, the patients and the therapist will not be blinded to the group allocation, because the protocol of the intervention prevents performing a real placebo with the CG. However, we will try to ensure the impartiality of the results, since the assessments and the statistical analysis will be carried out by a blinded researcher. Another limitation is the use of a self-reported measure to assess cognitive functioning as a primary outcome (the FACT-Cog scale), which could affect the effectiveness of the intervention because of the expectation bias in the EG. Nevertheless, we selected this measure based on previous study and for being the reference instrument for measuring CRCI in the oncological population [47], and the inclusion of objective cognitive measures as secondary outcomes would allow the data to be compared as recommended [48].

Regarding clinical implications of this study, there is a lack of treatment options to alleviate and to prevent CRCI. Non-pharmacological interventions, concrete cognitive training and cognitive-behavioral training programs have been gaining more interest as options that may be effective in managing this adverse effect. The current study suggests that an intervention that combines both approaches has the potential to prevent CRCI in women recently diagnosed with BC. If results of this study are positive, they could fill a knowledge gap in comprehensive oncology treatment. Given the lack of available therapies to prevent this problem, it is important to provide options with this objective and thus also achieve a better quality of life and daily living. Based on the current technological development and the economic benefits that telehealth has demonstrated in the oncological population, the consolidation of interventions such as the one proposed in this study may benefit not only the health and quality of life of patients but also reduce health-system costs, as it can be implemented as part of the healthcare system.

## 4. Conclusions

In conclusion, if the e-OTCAT program is effective, it will shed light on the benefits of this type of intervention in the prevention and mitigation of the side-effects of cancer and its neurotoxic treatments, specifically CRCI in BC patients. Similarly, it may contribute to supporting the potential advantages provided by telehealth approaches in oncology care.

## Figures and Tables

**Table 1:** Description of the e-OTCAT program in detail.

Weeks	Videoconference Sessions		Homework Exercises
	Day 1	Day 2	
W. 1	Explanation of work-plan	Cognitive training education: Attention	-20′ handbook exercises
			-20′ NeuroNation app
W. 2	Stress education and evaluation abilities training	Cognitive training implementation: Attention	-20′ handbook exercises
			-20′ NeuroNation app
			-Identify stressors throughout the week noting causes, consequences, emotions/thoughts/behaviours
W. 3	DB training	Cognitive training implementation: Attention	-20′ handbook exercises
			-20′ NeuroNation app
			-Self-registration to practice DB once daily
W. 4	PMR training	Cognitive training implementation: Attention	-20′ handbook exercises
			-20′ NeuroNation app
			-Self-registration to practice PMR daily
W. 5	Problem-solving training	Cognitive training education: Memory	-20′ handbook exercises
			-20′ NeuroNation app
			-Self-registration to practice PMR daily
			-Self-registration to practice problem solving
W. 6	Problem-solving continuation	Cognitive training implementation: Memory	-20′ handbook exercises
			-20′ NeuroNation app
			-Self-registration to practice PMR daily
			-Self-registration to practice problem solving
W. 7	Energy conservation and time management	Cognitive training implementation: Memory	-20′ handbook exercises
			-20′ NeuroNation app
			-Self-registration to practice PMR daily
			-Self-registration to practice energy conservation and time management
W. 8	DR training, I	Cognitive training implementation: Memory	-20′ handbook exercises
			-20′ NeuroNation app
			-Self-registration to practice DR-I
W. 9	DR training, II	Cognitive training education: Processing speed	-20′ handbook exercises
			-20′ NeuroNation app
			-Self-registration to practice DR-II
W. 10	DR training, III	Cognitive training implementation: Processing speed	-20′ handbook exercises
			-20′ NeuroNation app
			-Self-registration to practice DR-III
W. 11	Goals and personal needs: review of a chosen strategy	Cognitive training implementation: Processing speed	-20′ handbook exercises
			-20′ NeuroNation app
			-Self-registration to practice the chosen strategy
W. 12	Cognitive training implementation: Processing speed	Closure of the intervention and feedback	-

DP: Diaphragmatic Breathing; PMR: Progressive Muscle Relaxation; DR: Differential Relaxation.

## Data Availability

Not applicable.
